# Aerobic Denitrification Microbial Community and Function in Zero-Discharge Recirculating Aquaculture System Using a Single Biofloc-Based Suspended Growth Reactor: Influence of the Carbon-to-Nitrogen Ratio

**DOI:** 10.3389/fmicb.2020.01760

**Published:** 2020-08-04

**Authors:** Min Deng, Zhili Dai, Yeerken Senbati, Lu Li, Kang Song, Xugang He

**Affiliations:** ^1^State Key Laboratory of Freshwater Ecology and Biotechnology, Institute of Hydrobiology, Chinese Academy of Sciences, Wuhan, China; ^2^School of Materials Science and Chemical Engineering, Anhui Jianzhu University, Hefei, China; ^3^University of Chinese Academy of Sciences, Beijing, China; ^4^College of Fisheries, Huazhong Agricultural University, Wuhan, China

**Keywords:** aerobic denitrification, C/N ratio, nitrogen removal, qPCR, microbial community, function prediction

## Abstract

In this study, the effect of aerobic denitrification on nitrogen removal was investigated using two zero-discharge biofloc-based recirculating aquaculture systems with representative carbon-to-nitrogen (C/N) ratios of 15 (CN15) and 20 (CN20). Aquaculture wastewater, residual feed, and fish feces were treated in an aerated suspended growth reactor (SGR, dissolved oxygen > 5.0 mg L^–1^). Low toxic NH_3_ (<0.1 mg L^–1^) and NO_2_^–^-N (<0.5 mg L^–1^) concentrations and high NO_3_^–^-N (83.3%) and NO_2_^–^-N (100%) removal efficiencies were achieved in the fish tank and SGR of CN20, respectively. The nitrogen mass balances indicated that the gaseous nitrogen loss accounted for 72–75% of the nitrogen input. Illumina sequencing and quantitative polymerase chain reaction revealed that increasing the C/N ratio significantly increased the amount of aerobic denitrifying bacteria (*Dechloromonas*, *Rhodobacter*, *Flavobacterium*, and *Zoogloea*) and aerobic denitrifying functional genes (*napA*, *nirK*, and *nosZ*). Autotrophic *Nitrosomonas* was the dominant nitrifying bacteria in the CN15 system, and autotrophic (*Nitrosomonas*) and heterotrophic nitrifiers coexisted in the CN20 system. Moreover, the functional prediction analysis showed that the carbohydrate, energy, and amino acid metabolisms in the SGR of the latter increased. In conclusion, aerobic denitrification should widely exist in biofloc systems.

## Introduction

Aquaculture provided 80 million metric tons of fish for consumption in 2016 (FAO, 2018). Although cage and freshwater pond aquaculture produce the main aquatic products, industrial recirculating aquaculture systems (RAS) are expected to play a more important role in the future because of environmental and health concerns (Yogev and Gross, 2019). In aquaculture systems, 1–2 kg of feed is consumed to produce 1 kg of fish products. Approximately 75% of the nitrogen in the feed is released to the culturing environment as total ammonia nitrogen (TAN, 56.2%), feces (13.8%), and non-consumed feed (5%) (Jackson et al., 2003; Hari et al., 2006; Hu et al., 2012). However, TAN at low concentration can be toxic to fishes and therefore must be continuously removed (Yogev and Gross, 2019).

Traditional RASs consist of particulate solid waste removal and TAN treatment (nitrification) plants (Liu et al., 2019). Some RASs (almost 23.4% of aquaculture production companies) also contain denitrification plants to achieve zero water discharge (Badiola et al., 2012; Liu et al., 2019). However, waste treatment plants complicate RAS and demand long payback time (average of 8 years) for establishment and maintenance (Badiola et al., 2012). Moreover, because of the opposing demands for dissolved oxygen (DO) and organic matter, implementing waste treatment in RAS through conventional autotrophic nitrification and anaerobic denitrification in actual ecosystems is difficult. Biofloc-based suspended growth reactor (SGR) has been recently developed as substitute for the complicated treatment plants (Azim and Little, 2008; Liu et al., 2019; Yogev and Gross, 2019). Through organic carbon addition and continuous aeration, bioflocs are generated in the SGR and nitrogen is removed from the effluent of fish tanks (De Schryver and Verstraete, 2009; Liu et al., 2016, 2019; Yogev and Gross, 2019). Moreover, biofloc biomass can be consumed by fishes and thus recovers nitrogen (Azim and Little, 2008).

The performance measures of biofloc-based SGRs under different conditions, such as carbon sources, carbon-to-nitrogen (C/N) ratio, and aeration condition, were extensively studied (Liang et al., 2014; Luo et al., 2017b; Yogev and Gross, 2019). Biofloc systems usually require a C/N ratio of 15–20 (Avnimelech, 1999; Luo et al., 2017a). Microbial nitrogen assimilation was the most investigated process in biofloc systems because of the conversion of nitrogen waste into microbial protein, which can be used as fresh food for fishes (Gao et al., 2019). However, the nitrogen balance indicated that gaseous nitrogen loss, which is mainly caused by denitrification (NO_3_^–^ → NO_2_^–^ → NO → N_2_O → N_2_) (Hu et al., 2013), accounted for more than 60% of nitrogen input to the biofloc aquaculture system (Hu et al., 2014; Yogev and Gross, 2019); such loss can reduce the efficiency in nitrogen recovery. Given that high oxygen concentration (>5.0 mg L^–1^) in aquaculture systems significantly inhibits the activity of traditional anaerobic denitrification enzymes, denitrification was assumed to occur only in the inner anoxic region of the bioflocs (Nootong et al., 2011; Hu et al., 2013). However, a novel group of aerobic denitrifying bacteria, which can perform denitrification under aerobic condition, has been recently discovered in tidal flow constructed wetlands (Tan et al., 2020), sequencing batch biofilm reactor (Wang et al., 2017; Pan et al., 2020), airlift reactor (Ruan et al., 2016), and sequencing batch reactor (Alzate Marin et al., 2016) for wastewater treatment. Many aerobic denitrifiers are also capable of heterotrophic nitrification (Wang et al., 2017; Tan et al., 2020). However, the complex microbial process of nitrogen removal in biofloc systems was still unclear.

The periplasmic nitrate reductase (NAP) in aerobic denitrifiers can bear the inhibitory effect of the DO concentration (Sparacino-Watkins et al., 2014). Therefore, the existence of *napA* gene (encoding NAP) was widely used to indicate the aerobic denitrification ability of bacteria (Huang et al., 2013; Ji et al., 2015). Many bioinformatics tools were developed to analyze the function of bacteria using high-throughput sequencing data (Langille et al., 2013). PICRUSt was widely applied to gain deep insights into the microbial metabolic process of wastewater treatment reactors (Agrawal et al., 2019).

In this study, biofloc-based RASs (BRASs) containing different C/N ratios were established to treat real aquaculture wastes and achieve zero water discharge. First, the water quality in the fish tanks and the nitrogen removal efficiency of the SGRs were evaluated. Second, the relative abundance of *narG*, *napA*, *nirK*, *nirS*, and *nosZ* genes were measured using quantitative polymerase chain reaction (qPCR) to evaluate the denitrifying function. Subsequently, the bacterial communities were investigated through Illumina high-throughput sequencing to identify the main functional microbes. In addition, PICRUSt analysis was performed to obtain a comprehensive insight into the metabolism pathway.

## Materials and Methods

### System Setup

**FIGURE 1:**
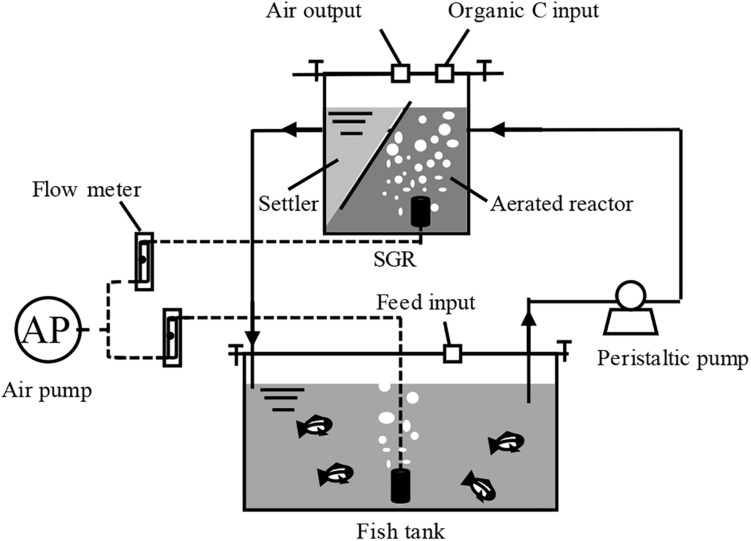
Schematic diagram of the zero-water discharge recirculating aquaculture system using a biofloc-based suspended growth reactor.

Two bench-scale BRASs were designed to achieve zero water discharge through a single SGR (Figure 1). Each system consists of a 40-L glass fish tank (30 L working volume) attached to a biofloc-based SGR. The SGR is composed of a 12-L continuously aerated reactor and a 5-L settler. Water, residual feeds, and feces from the fish tank were continuously pumped into the SGR at a rate of 0.5 L h^–1^ by a 5-W peristaltic pump (Kamoer Fluid Tech Co., Ltd., Shanghai, China). The generated biofloc in the settler returned to the aerated reactor because of gravity, and no biofloc biomass was removed during the entire experimental period. The treated water was recycled back to the fish tank. A 55-W air pump was used to perform aeration. The airflow rate was controlled using air flow meters (LZB-10, Senlod Co., Ltd., Nanjing, China) to maintain the high aerobic condition (approximately 5.0 mg L^–1^). The fish tanks and SGRs were sealed to minimize water evaporation.

### Experimental Design

Three weeks before the onset of the experiment, water containing bioflocs from a biofloc aquaculture system in Wuhan, China (30°32′48″ N, 114°21′0″ E), was added to the SGRs of two BRASs for microbe seeding. The water quality of the inoculated water (IW) is shown in Supplementary Table S1. The initial total suspended solid (TSS) for the two BRASs was 380 mg L^–1^. The microstructure of the bioflocs was loose, with a diameter of approximately 600 μm (Supplementary Figure S1). Crucians (*Carassius auratus* gibelio) were stocked at 20 kg m^–3^. The two systems were conducted under natural light. High aeration (DO > 5.0 mg L^–1^) and C/N ratio were set for the development of the biofloc during the 3-week start-up period (Table 1). The fishes were fed daily with 15 g of commercial feed (Xiamen Fwuso Industry Co., Ltd., Xiamen, China) containing 35% protein and 4% fat. Commercial tapioca starch (Hengshuifu Bridge Starch Co., Ltd., Hebei, China) was also introduced into the SGRs of the two BRASs to reach a basic C/N (w/w) ratio of 16 (Avnimelech, 1999). During the 3-week start-up period, the inorganic nitrogen concentrations in both BRASs stabilized (1.1 and 1.8 mg L^–1^ for TAN and NO_3_^–^-N, respectively, and daily change < 5%) for 1 week (Supplementary Figure S2). Then, the C/N ratio of the two systems was adjusted to 15 (CN15) and 20 (CN20). The details of the adjustment were provided in Supplementary Material. The systems were continuously run for another 35 days, and water samples from the different units (fish tank and SGR outlet) were collected every 2 days before feeding.

**TABLE 1:** Commercial feed feeding and adjusted C/N ratio in the two biofloc-based recirculating aquaculture systems.

	**Period (days)**	**Commercial feed (g)**	**Tapioca starch (g)**	**C/N**
Start-up*	21	15.1 ± 0.0	17.4 ± 0.6	16
CN15	35	15.2 ± 0.2	14.3 ± 0.4	15
CN20	35	15.4 ± 0.5	25.3 ± 0.6	20

*A 3-week start-up period has been conducted in the CN15 and CN20 systems for the development of biofloc and stable of inorganic nitrogen concentration in both systems.

### Water Quality Analysis

The TAN, NO_2_^–^-N, and NO_3_^–^-N concentrations were analyzed through spectrophotometry (Deng et al., 2018). The total nitrogen concentration of the fish, commercial feed, and biofloc were detected using multi N/C 3100 TOC analyzer (Analytik Jena AG, Jena, Germany). The nitrogen mass balance of the two systems was calculated using the method proposed by Hu et al. (2014). The temperature, DO, pH, and electrical conductivity were measured on-site daily using an HQ30d YSI meter (YSI Inc., Yellow Spring, OH, United States). The toxic NH_3_-N concentration in water was calculated in accordance with the approach of Emerson et al. (1975). The details are presented in Supplementary Material.

### Microbial Community Analysis

Three IW samples were collected at the beginning of the start-up period. Water samples from the fish tanks and SGRs of the two systems (CN15 and CN20) were collected in triplicate at the end of the experiment and mixed and marked as IW, T15, T20, R15, and R20 for the subsequent analysis. The DNeasy^®^ PowerSoil Kit (Qiagen, Hilden, Germany) was used to extract DNA. The primers 515F (5′-GTG CCA GCM GCC GCG GTA A-3′) and 806R (5′-GGA CTA CHV GGT WTC TAA T-3′) were used for bacterial 16S rRNA gene amplification. The library was constructed (Supplementary Material) and sequenced on an Illumina platform of Guangdong Magigene Biotechnology Co., Ltd. (Guangzhou, China) following the standard protocols. Phylogenetic classification and biodiversity analysis were conducted using the proposed technique of Deng et al. (2018). All raw sequencing data were stored in the Sequence Read Archive database (SRP267197).

### Functional Gene qPCR and Functional Profile Prediction

The abundance of the key denitrifying genes for nitrate reduction (*narG* and *napA*) (Bru et al., 2007), nitrite reduction (*nirS* and *nirK*) (Chen et al., 2017), and N_2_O reduction (*nosZ*) (Henry et al., 2006), along with the bacterial 16S rRNA genes (He et al., 2017) in two BRAS, were assessed by qPCR. The primers, reaction system, and annealing temperatures are presented in Supplementary Table S2. The functional potential of the microbial community was predicted on the basis of the 16S rRNA sequences using PICRUSt (Langille et al., 2013), and the functional predictions were assigned according to the standard method^1^. The Nearest Sequenced Taxon Index range (0.18–0.33) indicated relatively accurate predictions.

### Statistical Analysis

IBM SPSS Statistics 21 was used for the statistical analysis. The water quality and functional genes were measured in triplicate and expressed as mean ± SD. ANOVA was used to determine the significant differences at *p* < 0.05.

## Results and Discussion

### Water Quality in the Fish Tank

**FIGURE 2:**
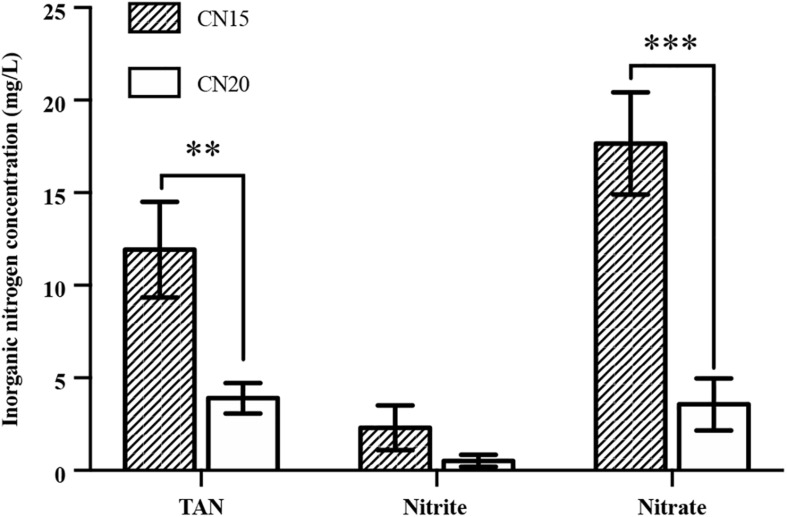
The concentrations of total ammonia nitrogen (TAN), nitrite, and nitrate in the fish tank of the CN15 and CN20 systems. ^∗∗^ *p* < 0.01; ^∗∗∗^ *p* < 0.001.

The experimental results show that the DO, temperature, and pH in the fish tanks were suitable for fish growth (Table 2). The TAN, NO_2_^–^-N, and NO_3_^–^-N concentrations in the fish tanks of CN15 and CN20 are shown in Figure 2. TAN, which comes from fish metabolism and uneaten feed and feces decomposition, is a major pollutant in the aquaculture system (Hu et al., 2012). Based on the daily feeding amount, the average TAN production in the fish tank was approximately 480 mg day^–1^ (Hu et al., 2012; Supplementary Material). TAN can be oxidized into NO_2_^–^-N and eventually NO_3_^–^-N in aquaculture systems because of nitrification (Liu et al., 2019). Without water discharge, total inorganic nitrogen (TIN) concentrations accumulate in BRAS. However, the final TIN in the CN20 and CN15 systems was less than 9.8 and 32.5 mg L^–1^, respectively, which indicated the high inorganic nitrogen removal potential in BRAS. Denitrification, which transforms NO_3_^–^-N and NO_2_^–^-N to N_2_O and N_2_ that are released to the air, is the main process that causes nitrogen loss in the BRAS (Hu et al., 2014). Given the high DO concentration (>5.0 mg L^–1^) in the fish tank and SGR, the denitrification in this study was processed under aerobic conditions. In addition, increasing the C/N ratio from 15 to 20 significantly decreased the TAN, NO_3_^–^-N, and NO_2_^–^-N concentrations in the fish tank by 67.3, 79.8, and 77.3%, respectively. This finding indicated that increasing the C/N ratio can improve the denitrification potential in BRAS.

**TABLE 2:** Dissolved oxygen, electrical conductivity (EC), pH, and temperature in the fish tanks (*n* = 35).

**Parameter**	**Mean**	**Standard error**	**Minimum**	**Maximum**
Dissolved oxygen (mg L^–1^)	5.2	0.5	4.4	6.0
EC (mS cm^–1^)	0.9	0.4	0.4	1.9
pH	7.6	0.2	7.2	8.0
Temperature (°C)	30.0	0.2	29.5	30.6

The NH_3_-N and NO_2_^–^-N in the fish tank should be strictly controlled because both are toxic to fishes even at low concentrations (Sun et al., 2012; Yogev and Gross, 2019). Therefore, the pollutants from the fish tanks were continuously treated in the SGR to avoid the accumulation of NH_3_-N and NO_2_^–^-N in the fish tanks in long or short term. The calculated NH_3_-N concentrations in the fish tanks of CN15 and CN20 were 0.30 and 0.10 mg L^–1^, respectively, which were significantly lower than the average acute toxicity concentration for 32 freshwater fish species (2.79 mg NH_3_ L^–1^) (Randall and Tsui, 2002). Although the CN15 system was NH_3_ toxicity-free, the high TAN concentration (average of 12 mg L^–1^) may still exert a negative effect on the immunity of the fishes during the long-term culturing period (Luo et al., 2014). The NO_2_^–^-N concentration in the CN20 system was below 1.0 mg L^–1^, whereas that in the CN15 system (2.3 mg L^–1^) exceeded the prescribed concentration for aquaculture systems (<1.5 mg L^–1^) (Yogev and Gross, 2019). The high NO_2_^–^-N concentration in the fish tank of the CN15 system was caused by the imbalance of the nitrifying bacteria in the system, which will be discussed in section “Microbial Community Analysis.”

### Nitrogen Removal Performance of SGR

The inorganic nitrogen removal efficiency of the SGRs with different C/N ratios is presented in Table 3. The TAN removal efficiency of the SGRs in both systems was lower than 25%, which slightly increased when the C/N ratio was increased from 15 to 20. A previous study reported the low TAN removal efficiency of a real SGR that treated aquaculture wastes (Liu et al., 2019). This unsatisfactory result can be attributed to the degradation of residual feeds and fish feces, which produced additional TAN in the SGR effluent (the details will be discussed in section “Microbial Functions”). Moreover, the high concentration of organic carbon in the SGR can inhibit the autotrophic nitrification activity (Ruan et al., 2016) and therefore reduce the TAN removal efficiency. In the CN20 system, the NO_2_^–^-N concentration in the fish tank ranged from 0.2 to 0.8 mg L^–1^, which was undetectable in the SGR effluent. The NO_2_^–^-N concentration in the SGR effluent of the CN15 system was higher than that in the influent; such high concentration was mainly due to the incomplete nitrification and denitrification because NO_2_^–^-N is the intermediate metabolite of both processes. The NO_3_^–^-N and NO_2_^–^-N removal efficiencies significantly increased when the C/N ratio was increased from 15 to 20 (*p* < 0.05), thereby indicating that a high amount of available carbon was beneficial to the aerobic denitrification in the SGR.

**TABLE 3:** Nitrogen removal efficiency of SGR in the CN15 and CN20 systems.

	**SGR influent (mg L^–1^)**		**SGR effluent (mg L^–1^)**		**Removal efficiency (%)**	
			
	**CN15**	**CN20**	**CN15**	**CN20**	**CN15**	**CN20**
TAN	11.9 ± 2.6	3.9 ± 0.8	9.9 ± 4.8	3.0 ± 0.4	16.8%	23.1%
NO_2_^–^-N	2.3 ± 1.2	0.5 ± 0.3	2.7 ± 1.6	0.0 ± 0.0	-17.4%*	100.0%
NO_3_^–^-N	17.7 ± 2.8	3.6 ± 1.4	14.8 ± 1.8	0.6 ± 0.8	16.4%	83.3%
TIN	31.9 ± 0.5	8.0 ± 1.7	24.1 ± 5.2	3.7 ± 1.1	11.4%	53.8%

*Negative value means NO_2_**^–^***-N concentration in effluent was higher than influent. TAN, total ammonia nitrogen; TIN, total inorganic nitrogen (TAN + NO_2_^–^-N + NO_3_^–^-N).*

The nitrogen mass balances of the CN15 and CN20 systems during the entire experimental period (day 0 to day 56) were evaluated (Table 4). Fish feed was the main nitrogen input to both systems. The CN15 system had a higher amount of inorganic nitrogen retained in water than the CN20 system because of the high TAN, NO_2_^–^-N, and NO_3_^–^-N concentrations in the water (Figure 2 and Table 3). Increasing the C/N ratio increased the increments in the fish and biofloc biomasses. The gaseous nitrogen loss accounted for 72–75% of the nitrogen input, which was similar to the findings of Hu et al. (2014) at a C/N ratio of 16 (76% gaseous nitrogen loss). The gaseous nitrogen loss in the CN20 system was slightly lower than that in the CN15 one, because the denitrifiers in the former were hungrier than those in the latter during the experimental period (the influent NO_2_^–^-N and NO_3_^–^-N were less than 0.5 and 5.0 mg L^–1^, respectively). These results indicated a high denitrification potential in the biofloc system because of the sufficient amount of organic carbon.

**TABLE 4:** Nitrogen (N) mass balance in the CN15 and CN20 systems.

**System**	**N input (g)**	**N output (g)**			
		**Inorganic N in water**	**Fish mass increase**	**Biofloc increase**	**Gaseous N loss**
CN15	47.5	1.4	7.0	3.7	35.4
CN20	47.9	0.3	8.1	5.1	34.4

### Microbial Community Analysis

The microbial community structures were further analyzed through high-throughput sequencing to investigate the difference in the inorganic nitrogen concentration and nitrogen removal efficiency in the BRASs with two different C/N ratios. A total of 97,884–338,198 high-quality sequencing tags were obtained, which corresponded to 1629–2136 operational taxonomic units (OTUs) (Table 5). The sequencing tags of IW were lower than those in the CN15 and CN20 systems. However, the obtained OTU and observed species were within the same range. The sequencing number for each sample was significantly higher than that in a previous research (Luo et al., 2017a), and the Chao1, Shannon, and Simpson indices of all samples reached stable values (Supplementary Figure S3), which indicated that the sequencing depth of the present study is sufficient to reflect the microbial community abundance and diversity. As shown in Table 5, the α-diversity (Chao1 richness and Shannon and Simpson diversity indices) of the samples in the CN20 system (T20 and R20) was higher than that in the CN15 system (T15 and R15). This result suggested that increasing the C/N ratio can enhance the microbial diversity of BRAS. The rich bacterial diversity in a system can result in a highly efficient nitrogen removal process (Ebeling et al., 2006). This inference is consistent with the lower TAN, NO_2_^–^-N, and NO_3_^–^-N concentrations of the CN20 system than the CN15 one (Figure 2).

**TABLE 5:** The α-diversity indices of microbial samples from inoculated water (IW) and the fish tanks and SGRs in the CN15 (T15 and R15) and CN20 (T20 and R20) systems.

**Samples**	**IW**	**T15**	**R15**	**T20**	**R20**
Sequencing tags	97884	338198	334833	306005	285577
OTU	1926	1906	1949	2136	1629
Observed species	1572	1036	1063	1425	1512
Chao1	2496	1753.5	1742.0	2161.8	1963.7
Shannon	5.97	4.71	4.84	6.27	5.97
Simpson	0.93	0.90	0.91	0.96	0.93

**FIGURE 3:**
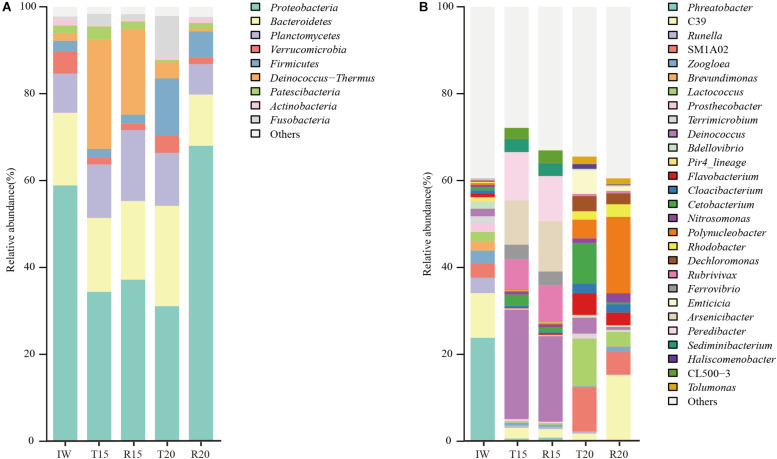
Microbial community structure in inoculated water (IW) and the fish tanks and SGRs of the CN15 (T15 and R15) and CN20 (T20 and R20) systems at phylum **(A)** and genus **(B)** levels. Only sequencing percentages >1% in at least one sample are shown.

The high-quality OTUs were annotated at phylum and genus levels, and those with a relative abundance higher than 1% are shown in Figure 3. *Proteobacteria* (58.9%) and *Bacteroidetes* (16.7%) were the predominant phyla in the IW and *Proteobacteria* (35.8 ± 2.0%), *Deinococcus*–*Thermus* (22.5 ± 3.9%), *Bacteroidetes* (17.6 ± 0.8%), and *Planctomycetes* (14.3 ± 2.8%) were the dominant ones in the fish tank and SGR in the CN15 system. The predominant phyla in the fish tank in the CN20 system included *Proteobacteria* (31.1%), *Bacteroidetes* (23.1%), *Firmicutes* (13.16%), and *Planctomycetes* (12.2%), whereas those in the SGR were *Proteobacteria* (68.0%) and *Bacteroidetes* (11.8%). The *Proteobacteria* in R20 was 2.0, 1.8, and 2.2 times higher than in T15, R15, and T20, respectively, which implied the high aerobic denitrification potential in the SGR of the CN20 system because aerobic denitrifying bacteria mainly belonged to this phylum (Chen et al., 2018).

**FIGURE 4:**
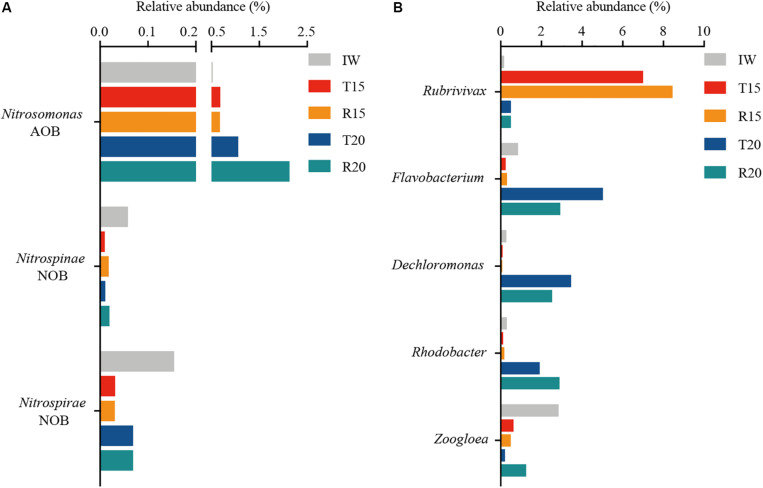
Relative abundance of **(A)** ammonia-oxidizing bacteria (AOB), nitrite-oxidizing bacteria (NOB), and **(B)** denitrifying bacteria in 16S rRNA gene-based microbial composition of inoculated water (IW), and the CN15 (T15 and R15) and CN20 (T20 and R20) systems.

Twenty-eight predominant genera (>1%) were retrieved at the genus level (Figure 3B). Different nitrifiers and denitrifiers, which play key roles in the biological nitrogen removal process, are displayed in Figure 4. For the nitrifiers, *Nitrosomonas* (0.7–2.1%) was the only detected ammonia-oxidizing bacteria (AOB), and its abundance in R20 was 3.1 times higher than in R15 (Figure 4A). The relative abundance of phyla *Nitrospinae* and *Nitrospirae*, which are nitrite-oxidizing bacteria (NOB), was extremely low (<0.1%). The AOB/NOB ratio in R15 and R20 was 14 and 24, respectively (Figure 4A). The relative abundance of NOB (autotrophic) in the CN15 and CN20 systems was lower than that in IW, which might because of the growth of heterotrophic bacteria in the two systems.

Azim and Little (2008) reported that *Nitrosomonas* is the dominant AOB species in aquaculture systems, and the addition of organic matter can enhance its abundance of *Nitrosomonas*. The high AOB/NOB ratio was consistent with the increase in NO_2_^–^-N concentration in the SGR effluent of the CN15 system (Table 3), suggesting that partial nitrification is the main process for the TAN removal in this system. Although the AOB/NOB ratio in R20 was 1.7-fold higher than that in R15, no NO_2_^–^-N accumulation was observed in the SGR of the CN20 system. To some extent, this may be the resulted of the significant increase in the aerobic denitrifiers in the SGR.

For the denitrifiers, *Rubrivivax* (phylum *Proteobacteria*) was the predominant genus (7.0–8.5%) in the CN15 system, and *Flavobacterium* (phylum *Bacteroidetes*), *Dechloromonas* (phylum *Proteobacteria*), and *Rhodobacter* (phylum *Proteobacteria*) were enhanced in the CN20 system (Figure 4B). *Zoogloea* (phylum *Proteobacteria*) dominated in IW and R20. The total abundance of abovementioned denitrifiers in the CN15 (8.1 and 9.5% for T15 and R15, respectively) and CN20 systems (11.1 and 10.1% for T20 and R20, respectively) was higher than that in IW (4.5%). The high AOB/NOB ratio and the increase in the NO_2_^–^-N concentration in the SGR effluent indicated the partial nitrification process in the CN15 system. *Rubrivivax* sp. (facultative–anaerobic denitrifying bacteria) was the main denitrifying bacteria in the CN15 system (87.6 ± 1.2% of the denitrifiers); such bacteria can transform NO_2_^–^-N to N_2_ without using NO_3_^–^-N as an electron acceptor (Zhang et al., 2016) because of the deficiency in nitrate reductase (Nagashima et al., 2012). These results implied that the partial nitrification and denitrification (PND, NH_4_^+^-N → NO_2_^–^-N → NO → N_2_O → N_2_) may be the main nitrogen removal process in the CN15 system.

The denitrification process in the CN20 system was mainly performed by aerobic denitrifying bacteria (*Dechloromonas*, *Rhodobacter*, *Flavobacterium*, and *Zoogloea*). *Dechloromonas* exhibited an excellent aerobic denitrification ability in tidal flow constructed wetland and was the dominant denitrifying and phosphorus-removing bacteria in the single-sludge sequencing batch reactor (Xu et al., 2018; Tan et al., 2020). *Rhodobacter* demonstrated enhancement in the single reactor for nitrogen removal under aerobic conditions (Chen et al., 2018). *Flavobacterium* (phylum *Bacteroidetes*) served as a complete aerobic denitrifier in the granular reactor (Pishgar et al., 2019). The amount of *Zoogloea*, which is correlated with biofloc formation (Weissbrodt et al., 2013) and facilitates aerobic denitrification (Huang et al., 2015; Pishgar et al., 2019), was increased in R20. In addition, *Dechloromonas*, *Flavobacterium*, and *Zoogloea* also displayed heterotrophic nitrification ability; therefore, it can conduct heterotrophic nitrification and aerobic denitrification (Huang et al., 2015; Pishgar et al., 2019; Tan et al., 2020). These results indicated the coexistence of autotrophic nitrification and heterotrophic nitrification in the CN20 system. Aerobic denitrifying bacteria (9.6–10.6% of the total bacteria) accounted for more than 95% of the denitrifying bacteria, thereby indicating that aerobic denitrification was the main process of denitrification in the CN20 system.

### Key Functional Genes of Denitrification

The key enzymes secreted by denitrifying bacteria, such as nitrate reductase (including NAR and NAP), nitrite reductase, and nitrous oxide reductase, are encoded using the corresponding functional genes. For nitrate reduction, the NAR encoded by *narG* is seriously inhibited by oxygen, whereas the NAP encoded by *napA* can be expressed under aerobic and anaerobic conditions (Sparacino-Watkins et al., 2014; Pan et al., 2020). The expression of the *napA* gene was often applied as an evidence of aerobic denitrification (Chen et al., 2018). The enzymes that catalyze the reduction of NO_2_^–^ to NO are encoded by *nirK* and *nirS* genes. Some *nirK* gene clusters in the denitrifying bacteria are similar to their homologs in the aerobic nitrifying bacteria in terms of sequence and organization and thus could be functional under aerated conditions (Cantera and Stein, 2007). The final step of denitrification is the catalysis by N_2_O reductase, which is encoded by the *nosZ* gene, such catalysis is uniform in the aerobic and anaerobic denitrifiers (Deng et al., 2019).

**FIGURE 5:**
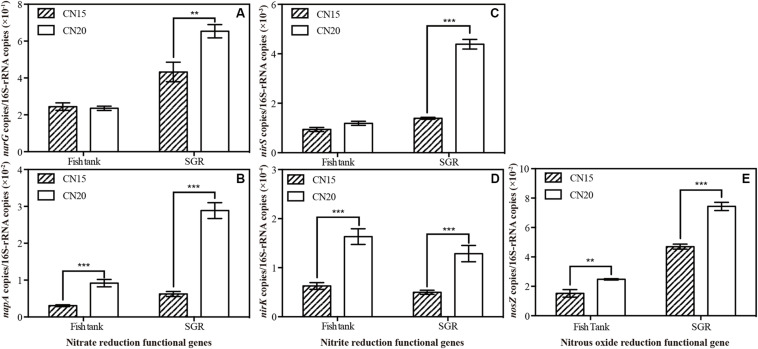
Relative abundance of denitrifying functional genes **(A)** *napA*/16S rRNA, **(B)** *nirG*/16S rRNA, **(C)** *nirK*/16S rRNA, **(D)** *nirS*/16S rRNA, and **(E)** *nosZ*/16S rRNA in the fish tanks and SGRs of the CN15 and CN20 systems. ^∗∗^*p* < 0.01; ^∗∗∗^*p* < 0.001.

To obtain a deep insight into the aerobic and anaerobic denitrification in BRAS, the relative abundance of the representative functional genes that encode the key denitrification enzymes was quantified through qPCR (Figure 5). For the nitrate reductase genes, the relative abundance of *napA* in T20 and R20 was 3.0 and 4.6 times higher than that in T15 and T20, respectively. A 1.5-fold increase was observed in the *narG* level in R20 (*p* < 0.05). For the nitrite reductase genes, the relative abundances of the *nirK* gene in T20 and R20 showed a 2.6-fold increase compared with those in T15 and R15. In addition, the relative abundance of the *nirS* gene was 3.1-fold higher in R20 than in R15. For the N_2_O reduction, increasing the C/N ratio from 15 to 20 significantly elevated the relative abundance of *nosZ* in the fish tank and SGR. Deng et al. (2019) reported that *napA*, *nirK*, and nosZ genes exhibited high oxygen tolerance and maintained aerobic denitrification. Hence, increasing the C/N ratio can provide additional electron donors for denitrification and increase the aerobic denitrification-related functional genes (*napA*, *nirK*, and *nosZ*) in the fish tank and SGR. The high amount of available organic carbon in the SGR of the CN20 system increased the heterotrophic biofloc biomass in SGR and provided the anaerobic environment for the expression of anaerobic denitrification-related functional genes (*narG* and *nirS*). These findings can explain the high nitrogen loss (N_2_ emission) in the biofloc-based aquaculture system (Hu et al., 2014; Yogev and Gross, 2019). Furthermore, these results indicated that increasing the C/N ratio of the biofloc-based SGR can significantly increase aerobic and anaerobic denitrification.

### Microbial Functions

**FIGURE 6:**
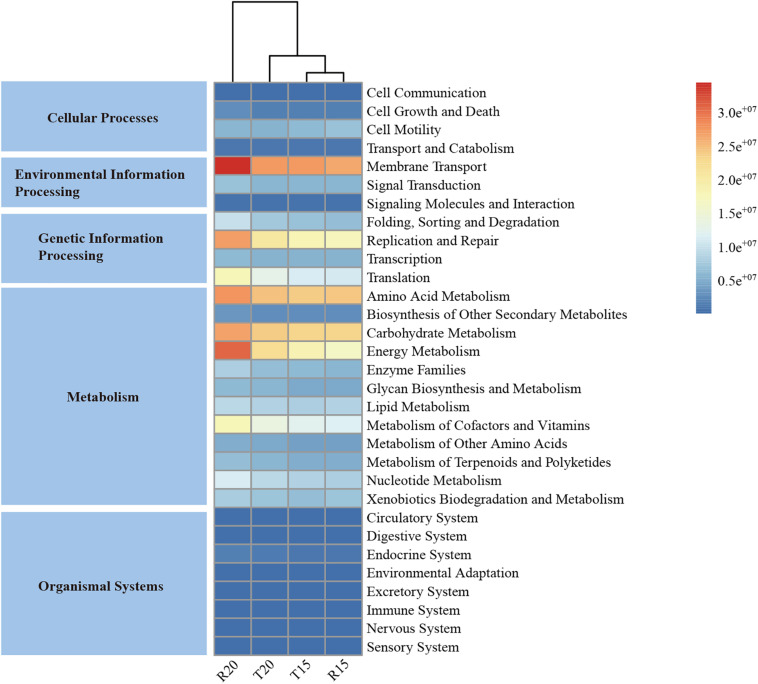
The metabolic pathways in different functional categories predicted from the 16S rRNA gene-based microbial compositions in the fish tanks and SGRs of the CN15 (T15 and R15) and CN20 (T20 and R20) systems using the PICRUSt algorithm.

The predicted functional potential of the microbial community in the CN15 and CN20 systems is illustrated in Figure 6. The functional potential of the microbial community in IW was not presented because of the relative low sequencing tags compared with those of the CN15 and CN20 systems. The PICRUSt results at the Kyoto Encyclopedia of Genes and Genomes Ortholog (KO) hierarchy level 1 demonstrated that metabolism (49.3–50.2%), genetic information processing (16.6–18.7%), and environmental information processing (12.7–13.5%) were the major microbial processes in both BRASs. At the KO hierarchy level 2, the most abundant functions included carbohydrate, energy, and amino acid metabolisms; membrane transport; and replication and repair, all of which were strengthened in R20.

The high C/N ratio in the SGR of the CN20 system was conducted through the addition of organic carbon source, which increased the carbohydrate metabolism (e.g., starch and sucrose metabolism and tricarboxylic acid cycle) in R20. This metabolism played an important role in degrading complex organic molecules (e.g., residual feeds, fish feces, and tapioca starch) into easily degradable substances to provide organic carbon sources for denitrifiers (Wei et al., 2018), which was consistent with the high energy metabolism (e.g., methane and nitrogen metabolisms) in R20 that provides energy for other microbial process. The increase in the membrane transport, replication, and repair indicated a high substance exchange (including nitrogen nutrient) in the bacterial cell, as well as a high bacterial activity in the SGR of the CN20 system. The increase in the amino acid metabolism increased the ammonia assimilation (Wu et al., 2017) and consequently improved the nitrogen removal efficiency in the CN20 system. Furthermore, the amino acid metabolism enabled bacteria to degrade residual feeds and feces and produce TAN, which may be the reason for the low TAN removal efficiency (<25%) in the SGR of the CN20 system (Table 3). These results indicated that increasing the C/N ratio can improve the nitrogen removal efficiency in SGR by increasing the carbohydrate, energy, and amino acid metabolisms.

## Conclusion

In this study, an RAS that uses a biofloc-based SGR for aquaculture waste treatment was constructed. Results showed that increasing the C/N ratio from 15 to 20 can significantly improve the water quality in the fish tank and enhance the nitrogen removal efficiency in the SGR by adjusting the microbial community structure and nitrogen removal pathway. PND was the main nitrogen removal pathway in the CN15 system. Autotrophic and heterotrophic nitrification coexisted in the CN20 system, and aerobic denitrification was the main denitrification process. The carbohydrate, energy, and amino acid metabolisms, along with membrane transport and replication and repair, were the key microbial processes that improved the nitrogen removal efficiency in the SGR of the CN20 system.

## Data Availability Statement

The datasets generated for this study can be found in the online repositories. The names of the repository/repositories and accession number(s) can be found at: https://www.ncbi.nlm.nih.gov/, SRP267197.

## Author Contributions

MD: conceptualization, software, data curation, and writing – original draft. ZD and YS: formal analysis. LL: writing – review and editing. KS: conceptualization and project administration. XH: conceptualization. All authors contributed to the article and approved the submitted version.

## Conflict of Interest

The authors declare that the research was conducted in the absence of any commercial or financial relationships that could be construed as a potential conflict of interest.

## Footnotes

1 http://picrust.github.io/picrust/

## References

[B1] Agrawal S. Kinh C. T. Schwartz T. Hosomi M. Terada A. Lackner S. (2019). Determining uncertainties in PICRUSt analysis – An easy approach for autotrophic nitrogen removal. *Biochem. Eng. J.* 152:107328 10.1016/j.bej.2019.107328

[B2] Alzate Marin J. C. Caravelli A. H. Zaritzky N. E. (2016). Nitrification and aerobic denitrification in anoxic-aerobic sequencing batch reactor. *Bioresour. Technol.* 200 380–387. 10.1016/j.biortech.2015.10.024 26512862

[B3] Avnimelech Y. (1999). Carbon/nitrogen ratio as a control element in aquaculture systems. *Aquaculture* 176 227–235. 10.1016/s0044-8486(99)00085-x

[B4] Azim M. E. Little D. C. (2008). The biofloc technology (BFT) in indoor tanks: water quality, biofloc composition, and growth and welfare of Nile tilapia (*Oreochromis niloticus*). *Aquaculture* 283 29–35. 10.1016/j.aquaculture.2008.06.036

[B5] Badiola M. Mendiola D. Bostock J. (2012). Recirculating aquaculture systems (RAS) analysis: main issues on management and future challenges. *Aquacul. Eng.* 51 26–35. 10.1016/j.aquaeng.2012.07.004

[B6] Bru D. Sarr A. Philippot L. (2007). Relative abundances of proteobacterial membrane-bound and periplasmic nitrate reductases in selected environments. *Appl. Environ. Microbiol.* 73 5971–5974. 10.1128/AEM.00643-07 17630306 PMC2074903

[B7] Cantera J. J. Stein L. Y. (2007). Molecular diversity of nitrite reductase genes (nirK) in nitrifying bacteria. *Environ. Microbiol.* 9 765–776. 10.1111/j.1462-2920.2006.01198.x 17298375

[B8] Chen H. Zhao X. Cheng Y. Jiang M. Li X. Xue G. (2018). Iron robustly stimulates simultaneous nitrification and denitrification under aerobic conditions. *Environ. Sci. Technol.* 52 1404–1412. 10.1021/acs.est.7b04751 29298384

[B9] Chen R. Deng M. He X. Hou J. (2017). Enhancing nitrate removal from freshwater pond by regulating carbon/nitrogen ratio. *Front. Microbiol.* 8:1712. 10.3389/fmicb.2017.01712 28943869 PMC5596099

[B10] De Schryver P. Verstraete W. (2009). Nitrogen removal from aquaculture pond water by heterotrophic nitrogen assimilation in lab-scale sequencing batch reactors. *Bioresour. Technol.* 100 1162–1167. 10.1016/j.biortech.2008.08.043 18842400

[B11] Deng M. Chen J. Gou J. Hou J. Li D. He X. (2018). The effect of different carbon sources on water quality, microbial community and structure of biofloc systems. *Aquaculture* 482 103–110. 10.1016/j.aquaculture.2017.09.030

[B12] Deng S. H. Li D. S. Yang X. Cai Q. Q. Peng S. Peng X. N. (2019). Novel characteristics on micro-electrolysis mediated Fe(0)-oxidizing autotrophic denitrification with aeration: Efficiency, iron-compounds transformation, N_2_O and NO_2_- accumulation, and microbial characteristics. *Chem. Eng. J.* 387:123409 10.1016/j.cej.2019.123409

[B13] Ebeling J. M. Timmons M. B. Bisogni J. J. (2006). Engineering analysis of the stoichiometry of photoautotrophic, autotrophic, and heterotrophic removal of ammonia–nitrogen in aquaculture systems. *Aquaculture* 257 346–358. 10.1016/j.aquaculture.2006.03.019

[B14] Emerson K. Russo R. C. Lund R. E. Thurston R. V. (1975). Aqueous ammonia equilibrium calculations: effect of pH and temperature. *J. Fish. Res. Board Can.* 32 2379–2383. 10.1139/f75-274

[B15] FAO (2018). *The State of World Fisheries and Aquaculture 2018 - Meeting the Sustainable Development Goals.* Rome: FAO.

[B16] Gao F. Liao S. Liu S. Bai H. Wang A. Ye J. (2019). The combination use of *Candida tropicalis* HH8 and *Pseudomonas* stutzeri LZX301 on nitrogen removal, biofloc formation and microbial communities in aquaculture. *Aquaculture* 500 50–56. 10.1016/j.aquaculture.2018.09.041

[B17] Hari B. Kurup B. M. Varghese J. T. Schrama J. W. Verdegem M. C. J. (2006). The effect of carbohydrate addition on water quality and the nitrogen budget in extensive shrimp culture systems. *Aquaculture* 252 248–263. 10.1016/j.aquaculture.2005.06.044

[B18] He X. Xu Y. Chen J. Ling J. Li Y. Huang L. (2017). Evolution of corresponding resistance genes in the water of fish tanks with multiple stresses of antibiotics and heavy metals. *Water Res.* 124 29–48. 10.1016/j.watres.2017.07.048 28738272

[B19] Henry S. Bru D. Stres B. Hallet S. Philippot L. (2006). Quantitative detection of the nosZ gene, encoding nitrous oxide reductase, and comparison of the abundances of 16S rRNA, narG, nirK, and nosZ genes in soils. *Appl. Environ. Microbiol.* 72 5181–5189. 10.1128/AEM.00231-06 16885263 PMC1538733

[B20] Hu Z. Lee J. W. Chandran K. Kim S. Khanal S. K. (2012). Nitrous oxide (N2O) emission from aquaculture: a review. *Environ. Sci. Technol.* 46 6470–6480. 10.1021/es300110x 22594516

[B21] Hu Z. Lee J. W. Chandran K. Kim S. Sharma K. Brotto A. C. (2013). Nitrogen transformations in intensive aquaculture system and its implication to climate change through nitrous oxide emission. *Bioresour. Technol.* 130 314–320. 10.1016/j.biortech.2012.12.033 23313675

[B22] Hu Z. Lee J. W. Chandran K. Kim S. Sharma K. Khanal S. K. (2014). Influence of carbohydrate addition on nitrogen transformations and greenhouse gas emissions of intensive aquaculture system. *Sci. Total. Environ.* 47 193–200. 10.1016/j.scitotenv.2013.09.050 24140689

[B23] Huang T. L. Zhou S. L. Zhang H. H. Bai S. Y. He X. X. Yang X. (2015). Nitrogen removal characteristics of a newly isolated indigenous aerobic denitrifier from oligotrophic drinking water reservoir. *Zoogloea* sp. N299. *Int. J. Mol. Sci.* 16 10038–10060. 10.3390/ijms160510038 25946341 PMC4463631

[B24] Huang X. Li W. Zhang D. Qin W. (2013). Ammonium removal by a novel oligotrophic *Acinetobacter* sp. Y16 capable of heterotrophic nitrification-aerobic denitrification at low temperature. *Bioresour. Technol.* 146 44–50. 10.1016/j.biortech.2013.07.046 23911816

[B25] Jackson C. Preston N. Thompson P. J. Burford M. (2003). Nitrogen budget and effluent nitrogen components at an intensive shrimp farm. *Aquaculture* 218 397–411. 10.1016/s0044-8486(03)00014-0

[B26] Ji B. Yang K. Zhu L. Jiang Y. Wang H. Zhou J. (2015). Aerobic denitrification: a review of important advances of the last 30 years. *Biotechnol. Bioproc. E.* 20 643–651. 10.1007/s12257-015-0009-0

[B27] Langille M. G. Zaneveld J. Caporaso J. G. McDonald D. Knights D. Reyes J. A. (2013). Predictive functional profiling of microbial communities using 16S rRNA marker gene sequences. *Nat. Biotechnol.* 31 814–821. 10.1038/nbt.2676 23975157 PMC3819121

[B28] Liang W. Luo G. Tan H. Ma N. Zhang N. Li L. (2014). Efficiency of biofloc technology in suspended growth reactors treating aquacultural solid under intermittent aeration. *Aquacul. Eng.* 59 41–47. 10.1016/j.aquaeng.2014.02.001

[B29] Liu W. Luo G. Tan H. Sun D. (2016). Effects of sludge retention time on water quality and bioflocs yield, nutritional composition, apparent digestibility coefficients treating recirculating aquaculture system effluent in sequencing batch reactor. *Aquacul. Eng.* 72-73 58–64. 10.1016/j.aquaeng.2016.04.002

[B30] Liu W. Tan H. Chen W. Luo G. Sun D. Hou Z. (2019). Pilot study on water quality regulation in a recirculating aquaculture system with suspended growth bioreactors. *Aquaculture* 504 396–403. 10.1016/j.aquaculture.2019.01.057

[B31] Luo G. Zhang N. Cai S. Tan H. Liu Z. (2017a). Nitrogen dynamics, bacterial community composition and biofloc quality in biofloc-based systems cultured *Oreochromis niloticus* with poly-β-hydroxybutyric and polycaprolactone as external carbohydrates. *Aquaculture* 479 732–741. 10.1016/j.aquaculture.2017.07.017

[B32] Luo G. Zhang N. Tan H. Hou Z. Liu W. (2017b). Efficiency of producing bioflocs with aquaculture waste by using poly-β-hydroxybutyric acid as a carbon source in suspended growth bioreactors. *Aquacul. Eng.* 76 34–40. 10.1016/j.aquaeng.2017.01.001

[B33] Luo G. Z. Gao Q. Wang C. H. Liu W. C. Sun D. C. Li L. (2014). Growth, digestive activity, welfare, and partial cost-effectiveness of genetically improved farmed tilapia (*Oreochromis niloticus*) cultured in a recirculating aquaculture system and an indoor biofloc system. *Aquaculture* 422–423, 1–7. 10.1016/j.aquaculture.2013.11.023

[B34] Nagashima S. Kamimura A. Shimizu T. Nakamura-Isaki S. Aono E. Sakamoto K. (2012). Complete genome sequence of phototrophic betaproteobacterium Rubrivivax gelatinosus IL144. *J. Bacteriol.* 194 3541–3542. 10.1128/JB.00511-12 22689232 PMC3434721

[B35] Nootong K. Pavasant P. Powtongsook S. (2011). Effects of organic carbon addition in controlling inorganic nitrogen concentrations in a biofloc system. *J. World Aquacult. Soc.* 42 339–346. 10.1111/j.1749-7345.2011.00472.x

[B36] Pan Z. Zhou J. Lin Z. Wang Y. Zhao P. Zhou J. (2020). Effects of COD/TN ratio on nitrogen removal efficiency, microbial community for high saline wastewater treatment based on heterotrophic nitrification-aerobic denitrification process. *Bioresour. Technol.* 301:122726. 10.1016/j.biortech.2019.122726 31927458

[B37] Pishgar R. Dominic J. A. Sheng Z. Tay J. H. (2019). Denitrification performance and microbial versatility in response to different selection pressures. *Bioresour. Technol.* 281 72–83. 10.1016/j.biortech.2019.02.061 30798089

[B38] Randall D. J. Tsui T. K. N. (2002). Ammonia toxicity in fish. *Mar. Pollut. Bull.* 45 17–23. 10.1016/s0025-326x(02)00227-8 12398363

[B39] Ruan Y. J. Deng Y. L. Guo X. S. Timmons M. B. Lu H. F. Han Z. Y. (2016). Simultaneous ammonia and nitrate removal in an airlift reactor using poly(butylene succinate) as carbon source and biofilm carrier. *Bioresour. Technol.* 216 1004–1013. 10.1016/j.biortech.2016.06.056 27343453

[B40] Sparacino-Watkins C. Stolz J. F. Basu P. (2014). Nitrate and periplasmic nitrate reductases. *Chem. Soc. Rev.* 43 676–706. 10.1039/c3cs60249d 24141308 PMC4080430

[B41] Sun H. Lü K. Minter E. J. A. Chen Y. Yang Z. Montagnes D. J. S. (2012). Combined effects of ammonia and microcystin on survival, growth, antioxidant responses, and lipid peroxidation of bighead carp *Hypophthalmythys nobilis* larvae. *J. Hazard. Mater.* 221–222 213–219. 10.1016/j.jhazmat.2012.04.036 22560242

[B42] Tan X. Yang Y. L. Li X. Zhou Z. W. Liu C. J. Liu Y. W. (2020). Intensified nitrogen removal by heterotrophic nitrification aerobic denitrification bacteria in two pilot-scale tidal flow constructed wetlands: influence of influent C/N ratios and tidal strategies. *Bioresour. Technol.* 302:122803. 10.1016/j.biortech.2020.122803 31981807

[B43] Wang J. Gong B. Wang Y. Wen Y. Zhou J. He Q. (2017). The potential multiple mechanisms and microbial communities in simultaneous nitrification and denitrification process treating high carbon and nitrogen concentration saline wastewater. *Bioresour. Technol.* 243 708–715. 10.1016/j.biortech.2017.06.131 28710998

[B44] Wei H. Wang L. Hassan M. Xie B. (2018). Succession of the functional microbial communities and the metabolic functions in maize straw composting process. *Bioresour. Technol.* 256 333–341. 10.1016/j.biortech.2018.02.050 29459320

[B45] Weissbrodt D. G. Neu T. R. Kuhlicke U. Rappaz Y. Holliger C. (2013). Assessment of bacterial and structural dynamics in aerobic granular biofilms. *Front. Microbiol.* 4:175. 10.3389/fmicb.2013.00175 23847600 PMC3707108

[B46] Wu J. Zhao Y. Qi H. Zhao X. Yang T. Du Y. (2017). Identifying the key factors that affect the formation of humic substance during different materials composting. *Bioresour. Technol.* 244 1193–1196. 10.1016/j.biortech.2017.08.100 28863988

[B47] Xu Z. Song L. Dai X. Chai X. (2018). PHBV polymer supported denitrification system efficiently treated high nitrate concentration wastewater: denitrification performance, microbial community structure evolution and key denitrifying bacteria. *Chemosphere* 197 96–104. 10.1016/j.chemosphere.2018.01.023 29334654

[B48] Yogev U. Gross A. (2019). Reducing environmental impact of recirculating aquaculture systems by introducing a novel microaerophilic assimilation reactor: modeling and proof of concept. *J. Clean. Prod.* 226 1042–1050. 10.1016/j.jclepro.2019.04.003

[B49] Zhang Q. Ji F. Xu X. (2016). Effects of physicochemical properties of poly-ε-caprolactone on nitrate removal efficiency during solid-phase denitrification. *Chem. Eng. J.* 283 604–613. 10.1016/j.cej.2015.07.085

