# Supplementary material for: Aerobic Denitrification Microbial Community and Function in Zero-Discharge Recirculating Aquaculture System Using a Single Biofloc-Based Suspended Growth Reactor: Influence of the Carbon-to-Nitrogen Ratio
Source: Front Microbiol. 2020 Aug 4;11:1760. doi: 10.3389/fmicb.2020.01760 (PMC7417430; doi:10.3389/fmicb.2020.01760)
Supplement: Supplementary file 1 [file Table_1.DOCX]

Supplementary Material

Aerobic denitrification microbial community and function in zero-discharge recirculating aquaculture system using a single biofloc-based suspended growth reactor: influence of the carbon-to-nitrogen ratio

**Min Deng^1^, Zhili Dai^2^, Bati Sen^1^, Lu Li^1^, Kang Song^1*^, Xugang He^3^**

^1^State Key Laboratory of Freshwater Ecology and Biotechnology, Institute of Hydrobiology, Chinese Academy of Sciences, Wuhan 430072, China

^2^School of Materials Science and Chemical Engineering, Anhui Jianzhu University, Hefei 230601, China

^3^College of Fisheries, Huazhong Agricultural University, Wuhan 430070, PR China

*** Correspondence:**Dr. Kang Song
sk@ihb.ac.cn

**1. Calculated methods**

The C/N ratio of commercial feed and starch added into the RAS was calculated according to the following equation:

C/N = (*F* × PC × 16% × 8.8 + *S* × 82.8% × 44.4%)/(*F* ×35% × 16%) (1)

Where *F* is the feeding rate (g day^-1^); PC is protein content in feed; 16% is the average nitrogen content of protein; 8.8 is the C/N ratio in the feeding commercial feed measured by Multi N/C 3100 TOC analyzer (Analytik Jena AG, Jena, Germany); *S* is the weight of daily tapioca starch addition; 82.8% is the carbohydrate in the commercial tapioca starch; and 44.4% is carbon content of starch.

The daily ammonia nitrogen generated in the water (to be treated) can be estimated using the following equation described by (Hu et al., 2012):

*P*_TAN_ = *F* × PC × 0.092 (2)

Where *P*_TAN_ is the production rate of total ammonia nitrogen (g day^−1^); *F* is the feeding rate (g day^−1^); and PC is protein content in feed (in fraction).

TAN contains un-ionized ammonia (NH_3_-N) and ionized ammonia (NH_4_^+^-N) and only NH_3_-N is toxic to fish. NH_3_-N concentrations were calculated using the general equation of bases (Emerson et al., 1975):

NH_3_ = (NH_3_ + NH_4_^+^)/[1+10^(p^*^K^*^a-pH)^] (3)

Where p*K*a is based on the equation developed by Emerson et al. (1975): p*K*a = 0.09018 + 2729.92/*T*, (*T* in ^◦^*K*).

**2. Library construction.**

DNA from all samples was amplified in triplicate by PCR for library construction. The 50-µL PCR mixture contained 25 µL of 2 × Premix Taq (Takara Biotechnology, Dalian Co. Ltd., China), 1µL of each primer (10 µM), 3µL of template DNA (20 ng µL^−1^). All mixtures had ddH_2_O added to a volume of 50µL and PCR were performed on a BioRad S1000 PCR thermal cycler (Bio-Rad Laboratory, CA, USA). The thermocycling steps were as follows: 94℃ for 5 min, followed by 30 cycles at 94℃ for 30 s, 52 ℃ for 30 s, 72 ℃ for 30s and a final extension step at 72 ℃ for 10 min. The library quality was assessed on the Qubit 2.0 Fluorometer (Thermo Fisher Scientific, MA, USA)

**3. Real-time quantitative PCR**

All specific primers for target genes were synthesized by Tyhygene Biotech Co., Ltd. (Wuhan, China). Each 20 µL reaction system consisted of 10 µL SYBR Premix (Takara, China), 0.5 µL DNA template, 0.5 µL each of forward and reverse primers, and 8.5 µL of sterile water. Forward and reverse primers for target genes and the annealing temperature are shown in Table S2. All plasmids containing the target genes were provided by Tyhygene Biotech Co., Ltd. (Wuhan, China) and subjected to 10 × serial dilution to serve as DNA templates for the construction of standard curves (R^2^ > 0.99). RT-qPCR was performed on a thermal cycler (CFX96, Bio-Rad, USA). Relative abundance (defined as the absolute number of genes normalized to the absolute number of ambient 16S-rRNA gene) was used in this study to compare the difference of denitrifying genes.


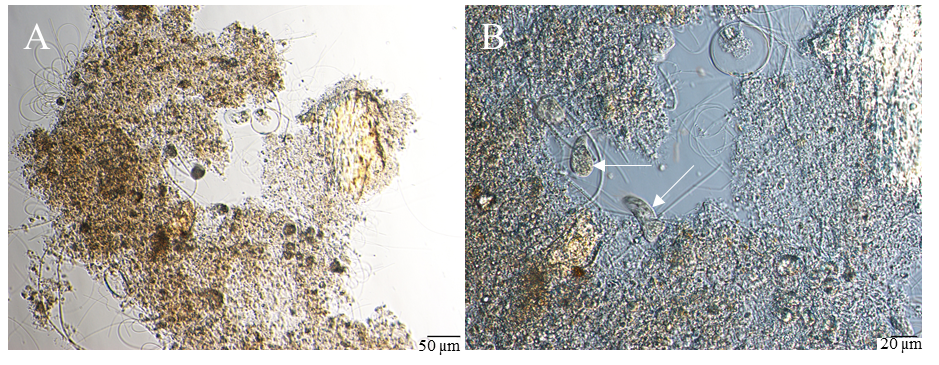


Figure S1. Morphology of the bioflocs in seeding water under the microscope (BX53, Olympus) with 20 × objective (A) and 40 × objective(B). White arrows show protozoa in the floc.


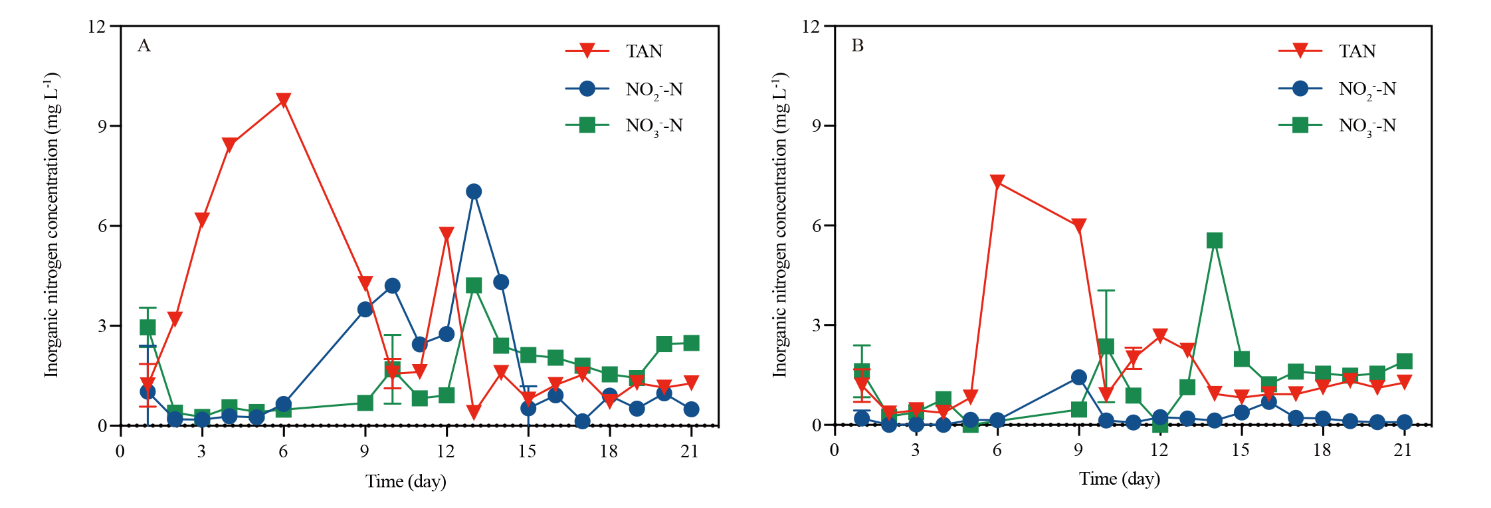


Fig. S2. Variation of total ammonia nitrogen (TAN), nitrite (NO_2_^−^-N) and nitrate (NO_3_^−^-N) concentration in (A) fish tanks and (B) suspended growth reactors (SGRs) during the 21-day start-up period.


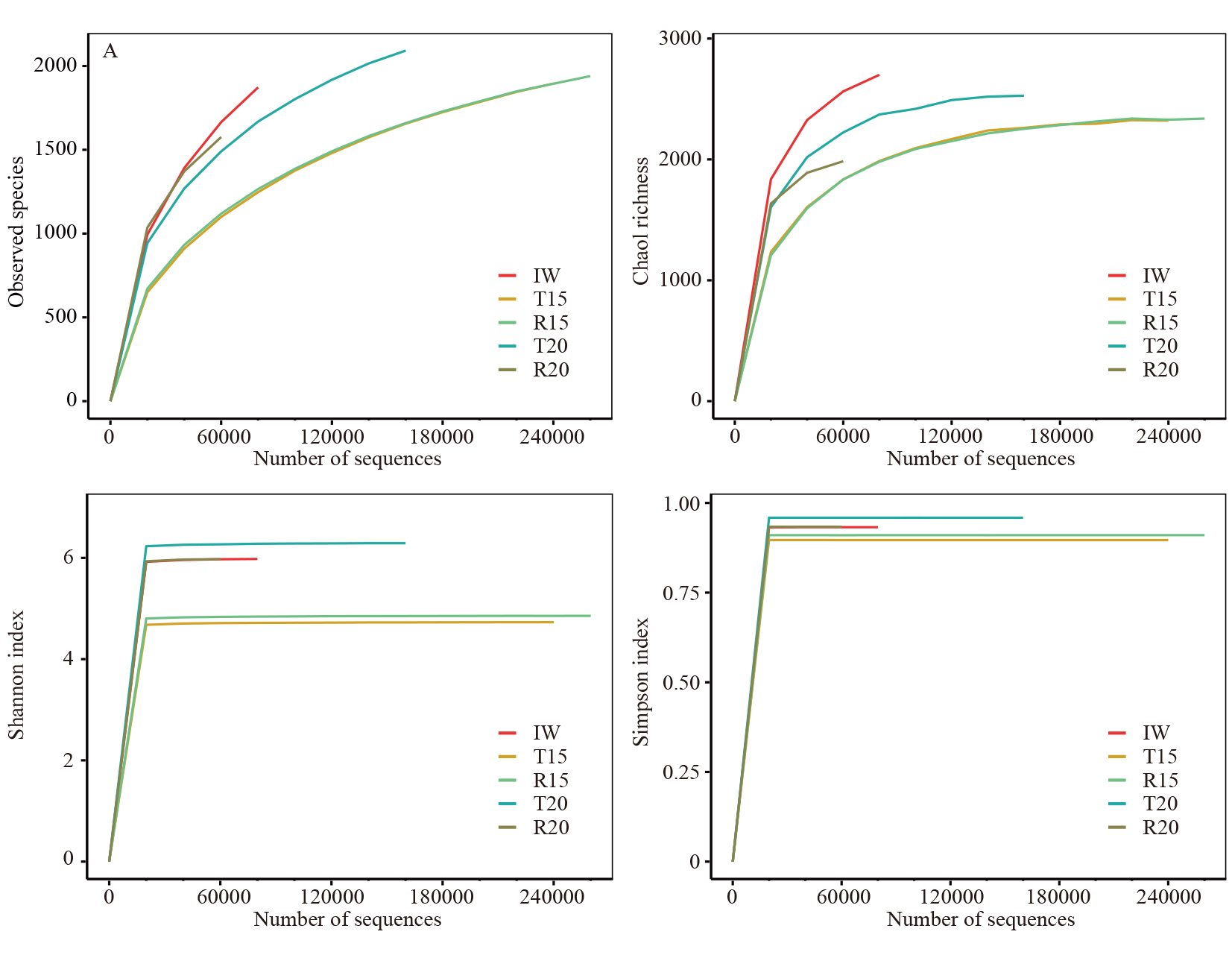


Figure S3. Rarefaction curves for microbial samples in inoculated water (IW) and the fish tanks and SGRs of the CN15 (T15, R15) and CN20 (T20, R20) systems. Rarefaction curves for observed species based on OTUs at 0.03 distance was calculated using Mothur (v1.27.0).

Table S1. Water quality in fish tanks and SGRs at the beginning of current experiment (n = 7).

| Parameters | Fish tanks | SGRs |
| --- | --- | --- |
| Total suspended solid (mg L^−1^)  Total ammonia nitrogen (mg L^−1^)  NH_3_-N (mg L^−1^)  Nitrite nitrogen (mg L^−1^)  Nitrate nitrogen (mg L^−1^) | 382 ± 2  1.2 ± 0.6  0.03 ± 0.01  0.5 ± 0.3  3.0 ± 0.6 | 385 ± 5  1.2 ± 0.5  0.03 ± 0.01  0.2 ± 0.3  1.6 ± 0.8 |

Table S2. Primers used for real-time quantitative PCR analysis of *narG*, *napA*, *nirK*, *nosZ* and 16S rRNA.

| Target gene | Primer | Primer sequence (5’ - 3’) | Annealing temperature (℃) | Amplification size (bp) | Reference |
| --- | --- | --- | --- | --- | --- |
| *narG*  *napA*  *nirK*  *nirS*  *nosZ*  16S rRNA | narG-f  narG-r  V17m  napA4r  nirKFlaCu  nirKR3Cu  nirScd3aF  nirS-R3cd  nosZ2F  nosZ2R  16SrRNA-F  16SrRNA-R | TCGCCSATYCCGGCSATGTC  GAGTTGTACCAGTCRGCSGAYTCSG  TGGACCATGGGCTTCAACC  ACCTCGCGCGCGGTGCCGCA  ATCATGGTSCTGCCGCG  GCCTCGATCAGRTTGTGGTT  GTSAACGTSAAGGARACSGG  GASTTCGGRTGSCTCTTGA  CGCRACGGCAASAAGGTSMSSGT  CAKRTGCAKSGCRTGGCAGAA  CGGTGAATACGTTCYCGG  GGHTACCTTGTTACGACTT | 58  61  55  55  60  55 | 173  152  473  426  276  142 | Bru et al., 2007  Bru et al., 2007  Chen et al., 2017  Chen et al., 2013  Henry et al. 2006  He et al. 2017 |

**Reference**

1. Bru, D., Sarr, A., Philippot, L., 2007. Relative abundances of proteobacterial membrane-bound and periplasmic nitrate reductases in selected environments. Appl. Environ. Microbiol. 73(18), 5971-5974.
2. Chen, R., Deng, M., He, X., Hou, J., 2017. Enhancing nitrate removal from freshwater pond by regulating carbon/nitrogen ratio. Front. Microbiol. 8, 1712.
3. He, X., Xu, Y., Chen, J., Ling, J., Li, Y., Huang, L., Zhou, X., Zheng, L., 2017. Evolution of corresponding resistance genes in the water of fish tanks with multiple stresses of antibiotics and heavy metals. Water Res. 124, 29-48.
4. Henry, S., Bru, D., Stres, B., Hallet, S., Philippot, L., 2006. Quantitative detection of the *nosZ* gene, encoding nitrous oxide reductase, and comparison of the abundances of 16S rRNA, *narG*, *nirK*, and *nosZ* genes in soils. Appl. Environ. Microbiol. 72, 5181-5189.
5. Hu, Z., Lee, J.W., Chandran, K., Kim, S., Khanal, S.K. (2012). Nitrous oxide (N2O) emission from aquaculture: a review. Environ. Sci. Technol. 46, 6470-6480. doi: 10.1021/es300110x
